# Memory T‐Cell Phenotype in Cutaneous T‐Cell Lymphoma Is Modified by Germline Gene Gametocyte Specific Factor 1

**DOI:** 10.1111/exd.70123

**Published:** 2025-05-14

**Authors:** Amelia Martínez Villarreal, Jennifer Gantchev, Pingxing Xie, Philippe Lefrançois, Brandon Ramchatesingh, Ivan V. Litvinov

**Affiliations:** ^1^ Faculty of Medicine and Health Sciences, Research Institute of the McGill University Health Centre McGill University Montreal Quebec Canada; ^2^ Division of Experimental Medicine, Faculty of Medicine and Health Sciences McGill University Montreal Quebec Canada; ^3^ Department of Neurosurgery Brigham and Women's Hospital, Harvard Medical School Boston Massachusetts USA; ^4^ Division of Dermatology, Faculty of Medicine and Health Sciences McGill University Montreal Quebec Canada; ^5^ Lady Davis Institute for Medical Research Jewish General Hospital, McGill University Montreal Quebec Canada

**Keywords:** cutaneous T‐cell lymphoma (CTCL), gametocyte specific factor 1 (GTSF1), prognostic biomarker, retrotansposons, Th2

## Abstract

Cutaneous T‐cell lymphoma (CTCL) is a heterogeneous group of lymphoproliferative disorders characterised by skin infiltration by malignant memory T cells. While most patients will present with an indolent disease, others will follow a highly aggressive clinical course. Currently, defining disease prognosis remains challenging. Ectopic expression of gametocyte‐specific factor 1 (GTSF1) has emerged as a potential prognostic biomarker. However, its contribution to CTCL carcinogenesis remains unknown. Here, we report that GTSF1 contributes to carcinogenesis by partially modifying the memory/effector phenotype of the malignant T cells. GTSF1 knockdown in CTCL cells led to T‐cell activation and production of IFNγ and TNFα. Advanced stages of the disease are associated with decreased production of these cytokines. Notably, we show that patients classified with high expression of GTSF1 are associated with a worse disease prognosis. Taken together, our findings indicate that GTSF1 expression in CTCL cells allows them to acquire memory T‐cell phenotype. Malignant memory T cells have a decreased production of immune‐responsive cytokines, leading to a diminished immune response and disease progression. GTSF1 is an important candidate as a prognostic biomarker. Furthermore, understanding the specific function of GTSF1 might help develop novel targeted treatment options for CTCL patients.

## 1 Introduction

Cutaneous T‐cell lymphoma (CTCL) is a rare and heterogeneous group of malignancies, characterised by the skin infiltration by malignant memory T cells [1, 2]. Life expectancy in the early stages is similar to a healthy control population; in contrast, life expectancy in advanced stages is less than ~2–3 years [3]. Approximately 25%–30% of patients with early disease progress to advanced stages [4]. Therefore, it is imperative to identify patients who have a higher risk of progression. Current prognostic factors are mainly based on clinical evaluation and certain pathological parameters (e.g., large cell transformation) [4, 5]. However, their usefulness remains limited [6]. Ectopic expression of gametocyte‐specific factor 1 (GTSF1) has been consistently reported to be associated with an aggressive disease course [7, 8, 9, 10, 11, 12, 13]. Therefore, GTSF1 has emerged as a potential prognostic biomarker.

High GTSF1 expression has been identified in patient samples with advanced stage disease (i.e., ≥ stage IIB) compared to patients with benign dermatoses and normal skin [7, 8, 9, 10, 12, 13, 14]. GTSF1 is essential for spermatogenesis and suppression of transposons. In mouse germline cells, it participates in a small RNA‐guided silencing system called the piRNA pathway. This pathway identifies active transposons and recruits the silencing machinery [15]. In that model, GTSF1 grasps and stabilises piRNAs during their maturation [16]. Considering that the reactivation of transposons has been associated with carcinogenesis [17], the expression of GTSF1 is highly relevant to CTCL carcinogenesis. However, its role remains to be elucidated.

CTCL is a highly heterogeneous entity both at the clinical and at the molecular levels [18, 19]. Clinical features in different variants include erythematous patches/plaques, violaceous tumours, loss of pigmentation, single/multiple lesions, poikiloderma, skin atrophy and a plethora of other symptoms [20]. The diverse clinical presentations highlight the heterogeneity of this malignancy. In addition, molecular variability between patients displaying the same variant, between different lesions of a single patient and even between different time points of a single lesion has been reported [10, 21, 22].

Progression has been associated with a shift of the predominant cytokines expressed in skin lesions [23]. In early stages, affected skin displays a Th1 immune‐responsive cytokine profile [24]. This profile is defined by high expression of STAT4 and production of IFNγ and TNFα. In late stages, patients present a polarisation towards a Th2 immune phenotype, characterised by the expression of IL‐4 and IL‐5 [13, 25, 26]. The mechanisms behind this cytokine profile shift and progression remain to be elucidated.

Here, we aimed to understand the contribution of GTSF1 to CTCL carcinogenesis and its impact on the phenotype of the malignant T cells. We reasoned that a detailed understanding of GTSF1 can help us discern its potential as a prognostic biomarker. Using clinical data and in vitro cell line models, we studied its role in CTCL. We observed that GTSF1 expression is associated with a memory T‐cell phenotype. In our in vitro work, GTSF1 knockdown leads to T‐cell activation and increased expression of the immune‐responsive cytokines, IFNγ and TNFα. This is mirrored in our clinical data evaluation, where high GTSF1 expression was associated with a worse clinical prognosis. We posit that high expression of GTSF1 in CTCL patients can be used as a progression biomarker of aggressive disease and unfavourable tumour microenvironment. Furthermore, GTSF1 contributes to the maintenance of a memory T cell phenotype, impacting the immune responsiveness of these patients.

## 2 Methods

### 2.1 Patients and Samples

All patients were enrolled in this study with written informed consent and in accordance with the Declaration of Helsinki. Samples were obtained after the approval from The Ottawa Hospital Research Institute (REB study #20150896‐01H), Research Institute of the McGill University Health Centre and affiliated hospitals (REB study #A09‐M81‐10A) and Laval University (REB study # 2011HES‐22 808). The patient cohort has been previously described [9]. Samples were obtained by punch biopsy of the lesional skin and processed into formalin‐fixed, paraffin‐embedded (FFPE) tissue blocks.

### 2.2 Cell Culture

All cell lines are human. Mac2A, MyLa 2000 (herein referred as MyLa), PB2B, HuT 78 and N/TERT‐1 are male‐derived cell lines, while SZ4 and Calu‐6 are female‐derived cell lines. Mac2A (RRID:CVCL_H637), MyLa (RRID:CVCL_8328), PB2B and SZ4 were obtained from Dr. K. Kaltoft and Dr. N. Ødum (University of Copenhagen, Copenhagen, Denmark). N/TERT‐1 (RRID:CVCL_CW92) was obtained from Dr. J. Rheinwald (Harvard Medical School, Boston, USA). Calu‐6 (ATCC Cat# HTB‐56 RRID:CVCL_0236) and HuT78 (ATCC Cat# TIB‐161 RRID:CVCL_0337) were purchased from the ATCC. All cell lines were cultured in their recommended media with the recommended percentage of Fetal Bovine Serum (FBS; Gibco Cat# 12484028) and 1% penicillin–streptomycin (p‐s; Gibco Cat# 15140122). Cells were maintained at 37°C in a humidified incubator containing 5% CO_2_.

### 2.3 Immunohistochemistry

To evaluate the expression of GTSF1 in CTCL samples, we performed immunohistochemistry staining using the Bond III system (Leica) with GTSF1 antibody (Abnova Cat# PAB23356 RRID:AB_11125113) and HRP‐conjugated compact polymer system and DAB (3, 3′‐diaminobenzidine). Slides were counterstained with haematoxylin. Slides were scanned with Aperio AT Turbo system (Leica).

### 2.4 Lentiviral shRNA‐Mediated Knockdown

To perform shRNA‐mediated knockdown of GTSF1, we transduced cell lines with a lentiviral vector. GIPZ Lentiviral shRNA vectors were purchased from Horizon Discovery: Clone V2LHS_24307 (Cat# VGH5518‐200211965), clone V3LHS_304723 (Cat# VGH5518‐200265235) and clone V3LHS_304726 (Cat# VGH5518‐200299947). The GIPZ non‐silencing vector (Cat# RHS4348) was used as a negative control. Transduction was performed with polybrene (Millipore Sigma Cat# TR‐1003‐G). Cells were selected with puromycin followed by GFP‐sorting with a BD FACSAria Fusion (BD Biosciences). Clone V2LHS_24307 was selected to perform all experiments.

### 2.5 RT‐qPCR Analysis of Gene Expression

To evaluate RNA expression levels, we performed RT‐qPCR. RNA was isolated with a RNeasy Mini Kit (Qiagen Cat# 74104) following the manufacturer's protocol. RNA was converted into cDNA with the iScript Advanced cDNA Kit for RT‐qPCR (Bio‐rad Cat#1725038). Gene expression levels were evaluated with qPCR using SsoAdvanced Universal SYBR Green Supermix (Bio‐rad Cat# 1725274) with the CFX Connect Real‐Time PCR Detection System (Bio‐rad). The housekeeping genes *GAPDH* or *ACTB* were used for standardisation following the Delta–Delta Ct Method.

### 2.6 Dual‐Luciferase Retrotransposition Assay

To evaluate the reactivation of retrotransposons, we performed an assay based on dual‐luciferase measurement. Plasmids pYX014, pYX015 and pYX017 were obtained from Dr. Wenfeng An (South Dakota State University, USA) [27]. Cells were transiently transfected with Cell Line Nucleofector Kit V (Lonza Cat# VCA‐1003) and Lonza Nucleofector Transfection 2b Device (Lonza RRID:SCR_022262). Luminescence was detected according to the manufacturer's protocol of Dual‐Luciferase Reporter Assay System (Promega Cat # E1910) with an Infinite M200 PRO (Tecan RRID:SCR_019033) microplate reader. Each firefly luminescence value was divided by its corresponding *Renilla* value to correct for transfection efficiency and cell survival. Ratios corresponding to pYX015 were used as normalisation factors.

### 2.7 Cell Proliferation Assay

Cell proliferation was measured using the Vi‐CELL XR (Beckman Coulter RRID:SCR_019664) every 24 h until 144 h.

### 2.8 Apoptosis Assay

To evaluate cell survival, we performed annexin V/PI staining. Cells were resuspended in annexin V binding buffer, stained with propidium iodide (PI; Invitrogen Cat# P1304MP) and 1 μL of annexin V, Alexa Fluor 647 conjugate (Invitrogen Cat# A23204). Cells were acquired in a BD FACSCanto II system (BD Biosciences RRID:SCR_018056) with BD FACSDiva Software (Version 8.0.2 BD Biosciences RRID:SCR_001456). Data analysis was performed using the FlowJo software (Version 10.9.0 BD Life Sciences RRID:SCR_008520).

### 2.9 Immunofluorescence

To evaluate proliferation, we performed immunofluorescence staining for Ki67. The following antibodies were used: Ki67 primary antibody (Invitrogen Cat# PA5‐16785 RRID:AB_11000602) and secondary Anti‐rabbit Alexa Fluor 594 Conjugate (Cell Signaling Cat# 8889 RRID:AB_2716249). Cover slips were mounted on slides with Fluoroshield with DAPI (Millipore Sigma Cat# F6057). Visualisation and photos were acquired with a Lumascope LS720 microscope (Etaluma). Positive cells were quantified with QuPath (version 0.4.4 RRID:SCR_018257).

### 2.10 Flow Cytometry Analysis of Cell Surface Markers

To evaluate changes in cell surface marker, we performed staining followed by flow cytometry analysis. Cells were stained with eBioscience Fixable Viability Dye eFluor 780 (Invitrogen Cat# 65‐0865‐14). The BD Horizon Brilliant Stain Buffer (BD Biosciences Cat# 563794) was used for staining with the fluorochrome‐conjugated antibody BV421‐CD25 (Biolegend Cat# 302630 RRID:AB_11126749). Cells were acquired in the BD FACSCanto II system (BD Biosciences) with BD FACSDiva Software (Version 8.0.2 BD Biosciences). Data analysis was performed using the FlowJo software (Version 10.9.0 BD Life Sciences).

### 2.11 Cytokine Membranes

To evaluate cytokine dysregulation, we tested cell culture supernatant with a Human Th1/Th2/Th17 Antibody Array (Abcam Cat# ab169809), as per the manufacturer's protocol. Detection was performed in a ChemiDoc MP Imaging System (Bio‐rad RRID:SCR_019037). Quantification was performed with the Image Lab Software (Version 6.1 Bio‐rad RRID:SCR_014210).

### 2.12 ELISA Assays

To quantify production of relevant cytokines, IFNγ (Invitrogen Cat# BMS228) and TNFα (Abcam Cat# ab181421), we performed ELISAs, as per the manufacturer's protocol. Absorbance values were measured with an Infinite M200 PRO (Tecan) microplate reader and corrected with the blank wells. The standard curve was plotted with the online Quest Graph Four Parameter Logistic (4PL) Curve Calculator (AAT Bioquest https://www.aatbio.com/tools/four‐parameter‐logistic‐4pl‐curve‐regression‐online‐calculator).

### 2.13 Extracellular Lactate Detection

To evaluate metabolic changes of memory/effector cells, we quantified lactate secretion using the Lactate‐Glo Assay Kit (Promega Cat# J5021), as per the manufacturer's protocol. Luminescence was recorded with an Infinite M200 PRO (Tecan) microplate reader and normalised to growth media alone.

### 2.14 Western Blotting

To evaluate protein expression, we performed western blotting with 4%–20% Mini‐PROTEAN TGX Stain‐Free Precast Gels (Bio‐rad Cat# 4568093 and 4568094), followed by transfer to 0.22 μm PVDF membranes (Bio‐rad Cat# 1704157 and 1704156). Overnight incubation at 4°C was carried out with the following primary antibodies: DDX4 (Abcam Cat# ab27591 RRID:AB_11139638), GAPDH (Thermo Fisher Scientific Cat# PA1‐987 RRID:AB_2107311), GTSF1 (Abcam Cat# ab262937), LINE‐1 Orf1p (Millipore Sigma Cat# MABC1152 RRID:AB_2941775), NFκB2 p100/p52 (Cell Signaling Cat# 4882 RRID:AB_10695537), PIWIL2 (Abcam Cat# ab181340), PIWIL4 (Abcam Cat# ab111714 RRID:AB_10887762), pSTAT3 Y705 (Cell Signaling Cat# 9145 RRID:AB_2491009), STAT3 (Cell Signaling Cat# 30835 RRID:AB_2798995), STAT4 (Cell Signaling Cat# 2653, RRID:AB_2255156), STAT5 (Cell Signaling Cat# 94205 RRID:AB_2737403), pSTAT6 (Cell Signaling Cat# 9361 RRID:AB_331595), STAT6 (Cell Signaling Cat# 5397 RRID:AB_11220421) and TDRD9 (Abcam Cat# ab118427). Secondary antibody incubation for 1 h was performed with the appropriate HRP‐conjugated secondary antibody: anti‐mouse IgG (Cell Signaling Cat# 7076 RRID:AB_330924) or anti‐rabbit IgG (Cell Signaling Cat# 7074 RRID:AB_2099233). For the chemiluminescence reaction, the Clarity Western ECL Substrate kit (Bio‐rad Cat# 1705061) was used, and detection was performed in a ChemiDoc MP Imaging System (Bio‐rad). Image processing was done with Image Lab Software (Version 6.1 Bio‐rad).

### 2.15 Bulk RNA‐Sequencing

To evaluate the transcriptomic changes, we performed bulk RNA‐Sequencing. RNA was isolated with an RNeasy Mini Kit (Qiagen Cat# 74104) following the manufacturer's protocol. Quality analysis, library preparation and sequencing were performed by Génome Québec.

### 2.16 Analysis of RNA‐Seq From Cell Lines

RNA‐Seq analysis [28, 29, 30, 31, 32] was performed by the Rnomics platform at the Université de Sherbrooke. DESeq2 (Version 1.34 RRID:SCR_015687) was used to identify Differentially Expressed Genes (DEGs) between SCR control and shGTSF1 knockdown conditions. Functional enrichment analysis was performed with DEGs at Log_2_ fold change ≥ 1 or Log_2_ fold change ≤ −1 and *p*‐adj < 0.05 using g:Profiler (Version e109_eg56_p17_1d3191d RRID:SCR_006809) [33, 34, 35]. The Venny 2.1 software was used to identify common DEGs. Gene lists used in heatmaps were accessed from KEGG Database [36].

### 2.17 Analysis of Publicly Available Datasets

For analysis of The Cancer Genome Atlas (TCGA), RNA‐Seq data from 33 cancers were downloaded from cBioPortal (RRID:SCR_014555) [37, 38, 39]. Mean Transcript Per Million (TPMs) were compared with Bayesian analysis using Markov Chain Monte Carlo with rjags (Version 4.3.0 RRID:SCR_017573) [40]. Multiple hypothesis testing adjustment was performed with the Bonferroni method.

For the CTCL dataset, RNA‐Seq data (GEO accession number: GSE168508) [41] were obtained along with clinical data [42, 43]. For survival analysis, patients were ranked based on *GTSF1* mRNA level of expression. The upper tertile was classified as high and the two lower tertiles were classified as low, as previously described [44]. Differential expression was evaluated with the Mann–Whitney test and survival analysis was performed with the Log‐rank (Mantel‐Cox) test.

### 2.18 Quantification and Statistical Analysis

Graphs and statistical analyses were performed using the software GraphPad Prism (Version 10.0.1 RRID:SCR_002798). Normality assumptions were tested with Shapiro–Wilk analysis and the F test. If normality assumptions were met, differences between means of three biological replicates were determined by a *t* test. In the case of multiple comparisons, the Mann–Whitney test was used with the Holm‐Šídák correction method. Error bars represent standard deviation, and statistical significance was considered at *p* < 0.05. Figures were created using Inkscape (Version 1.2 RRID:SCR_014479).

## 3 Results

**FIGURE 1:**
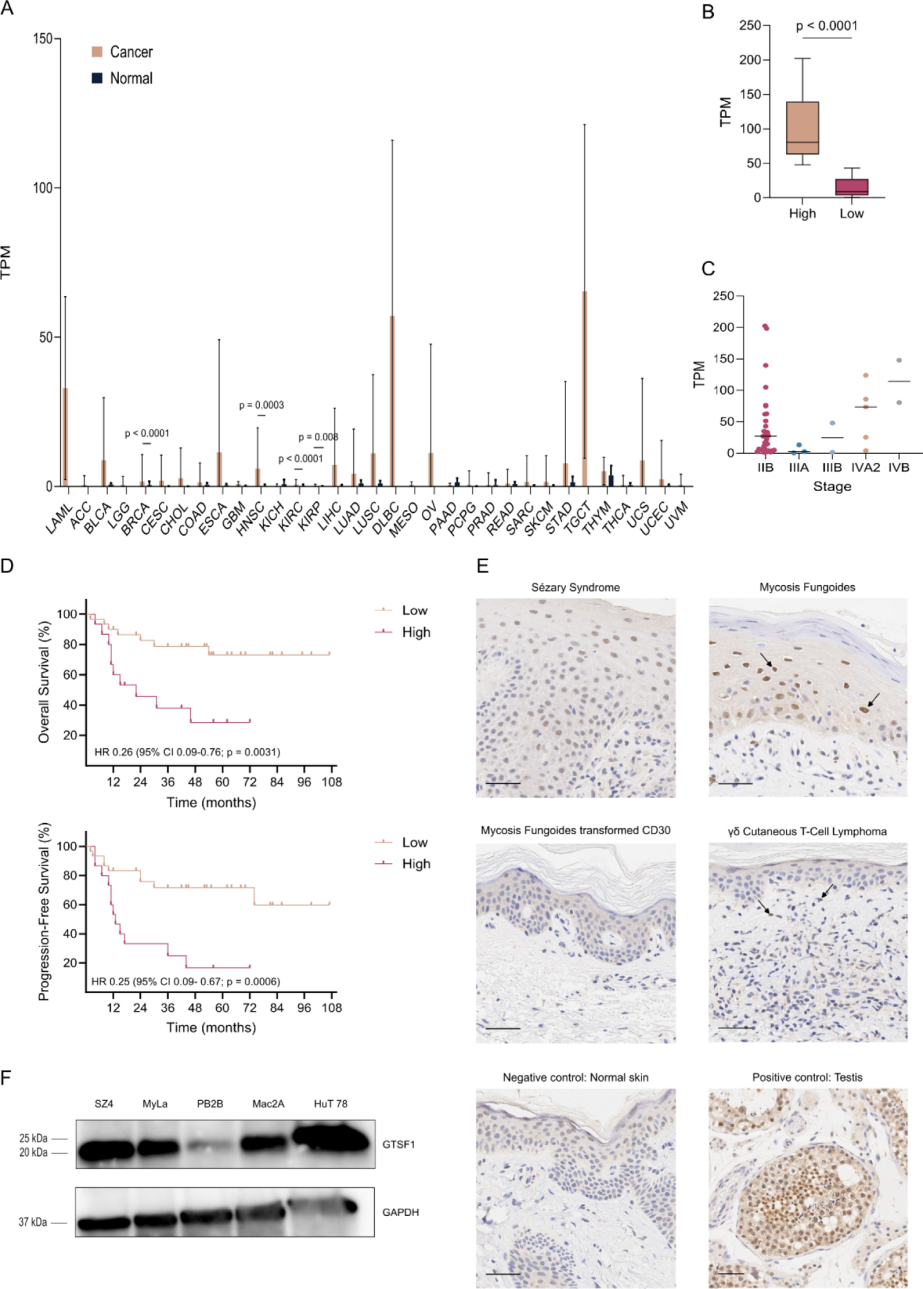
Heterogeneous GTSF1 expression in cancer and CTCL. (A) Relative *GTSF1* expression in Transcripts Per Million (TPM) for 33 cancer types (brown bars) and their normal counterparts (black bars) from The Cancer Genome Atlas (TCGA). Differential expression between cancer and normal adjacent tissue was evaluated with Markov Chain Monte Carlo and Bonferroni adjustment with *p* < 0.05. Data are presented as means ± SD. (B) Relative *GTSF1* expression in TPM in CTCL patients from GSE168508. Differential expression between high (brown bars) and low (pink bars) *GTSF1* was evaluated with Mann–Whitney test with *p* < 0.05, whiskeys represent minimum and maximum and lines in the middle of boxes represent the median. (C) Number of patients on each disease stage classified in high or low *GTSF1* expression from GSE168508. (D) Survival (Kaplan–Meier) plots of CTCL patients' disease outcomes: Overall Survival (top) and Progression‐Free Survival (bottom). Differential survival between high (brown bars) and low (pink bars) *GTSF1* expression groups was identified with Log‐rank (Mantel‐Cox) test with *p* < 0.05. (E) Representative immunohistochemistry of GTSF1 in skin biopsies from CTCL patients. Each panel represents a different patient. Arrows indicate nuclear expression of GTSF1 in pleomorphic epidermotropic lymphocytes. Negative control (normal skin) is presented bottom left and positive control (normal human testis) is presented bottom right. Scale bars represent 50 μm. (F) Western blot analysis of GTSF1 in CTCL cell lines. GAPDH was used as a loading control. ACC, adrenocortical carcinoma; BLCA, bladder urothelial carcinoma; BRCA, breast invasive carcinoma; CESC, cervical squamous cell carcinoma and endocervical adenocarcinoma; CHOL, cholangiocarcinoma; COAD, colon adenocarcinoma; DLBC, lymphoid neoplasm diffuse large B‐cell lymphoma; ESCA, oesophageal carcinoma; GBM, glioblastoma multiforme; HNSC, head and neck squamous cell carcinoma; KICH, kidney chromophobe; KIRC, kidney renal clear cell carcinoma; KIRP, kidney renal papillary cell carcinoma; LAML, acute myeloid leukaemia; LGG, brain lower grade glioma; LIHC, liver hepatocellular carcinoma; LUAD, lung adenocarcinoma; LUSC, lung squamous cell carcinoma; MESO, mesothelioma; OV, ovarian serous cystadenocarcinoma; PAAD, pancreatic adenocarcinoma; PCPG, pheochromocytoma and paraganglioma; PRAD, prostate adenocarcinoma; READ, rectum adenocarcinoma; SARC, sarcoma; SKCM, skin cutaneous melanoma; STAD, stomach adenocarcinoma; TGCT, testicular germ cell tumours; THCA, thyroid carcinoma; THYM, thymoma; UCEC, uterine corpus endometrial carcinoma; UCS, uterine carcinosarcoma; UVM, uveal melanoma.

To study the role of ectopic expression of GTSF1 in carcinogenesis, we evaluated gene expression data from TCGA. We compared the level of *GTSF1* mRNA expression between cancer and their normal adjacent tissue across 33 cancer types (Figure 1A). Our analysis demonstrated that *GTSF1* is significantly overexpressed in Breast Invasive Carcinoma (BRCA), Head and Neck Squamous Cell Carcinoma (HNSC), Kidney Renal Clear Cell Carcinoma (KIRC) and Kidney Renal Papillary Cell Carcinoma (KIRP). In addition, Acute Myeloid Leukaemia (LAML), Lymphoid Neoplasm Diffuse Large B‐cell Lymphoma (DLBC) and Testicular Germ Cell Tumours (TGCT) showed a high level of expression. For this set of malignancies, no normal adjacent tissue data were available. This highlights that *GTSF1* is heterogeneously ectopically expressed across different cancer types. Considering its ectopic expression has been consistently reported in CTCL, we hypothesised that this indicates a particular and specific role in carcinogenesis of this lymphoma.

To evaluate the impact ectopic expression of GTSF1 has on CTCL prognosis/disease outcome, we conducted a survival analysis with a publicly available dataset (GEO accession number: GSE168508) [41]. We ranked patients based on the level of *GTSF1* mRNA expression and classified them in high versus low [44] expression groups (Figure 1B). Interestingly, the two patients with the most advanced stage (IVB) were classified in the high *GTSF1* expression group (Figure 1C). Furthermore, patients in the high *GTSF1* expression group had decreased overall survival and accelerated disease progression compared to patients in the low expression group (Figure 1D), particularly patients at disease stage IIB (Figure S1A). To evaluate GTSF1 expression at the protein level, we performed immunohistochemical staining of skin biopsies from CTCL patients (Figures 1E and S1B). GTSF1 expression was detected in a heterogeneous pattern. Some samples demonstrated nuclear GTSF1 expression in pleomorphic epidermotropic lymphocytes (Figure 1E, arrows).

Next, we sought to determine whether CTCL patient‐derived cell lines expressed GTSF1. We performed RT‐qPCR and western blot analysis with commonly used CTCL cell lines. These cell lines represent the most common variants of CTCL [45] (Figures 1F and S1C). All cell lines expressed GTSF1 mRNA and protein.

**FIGURE 2:**
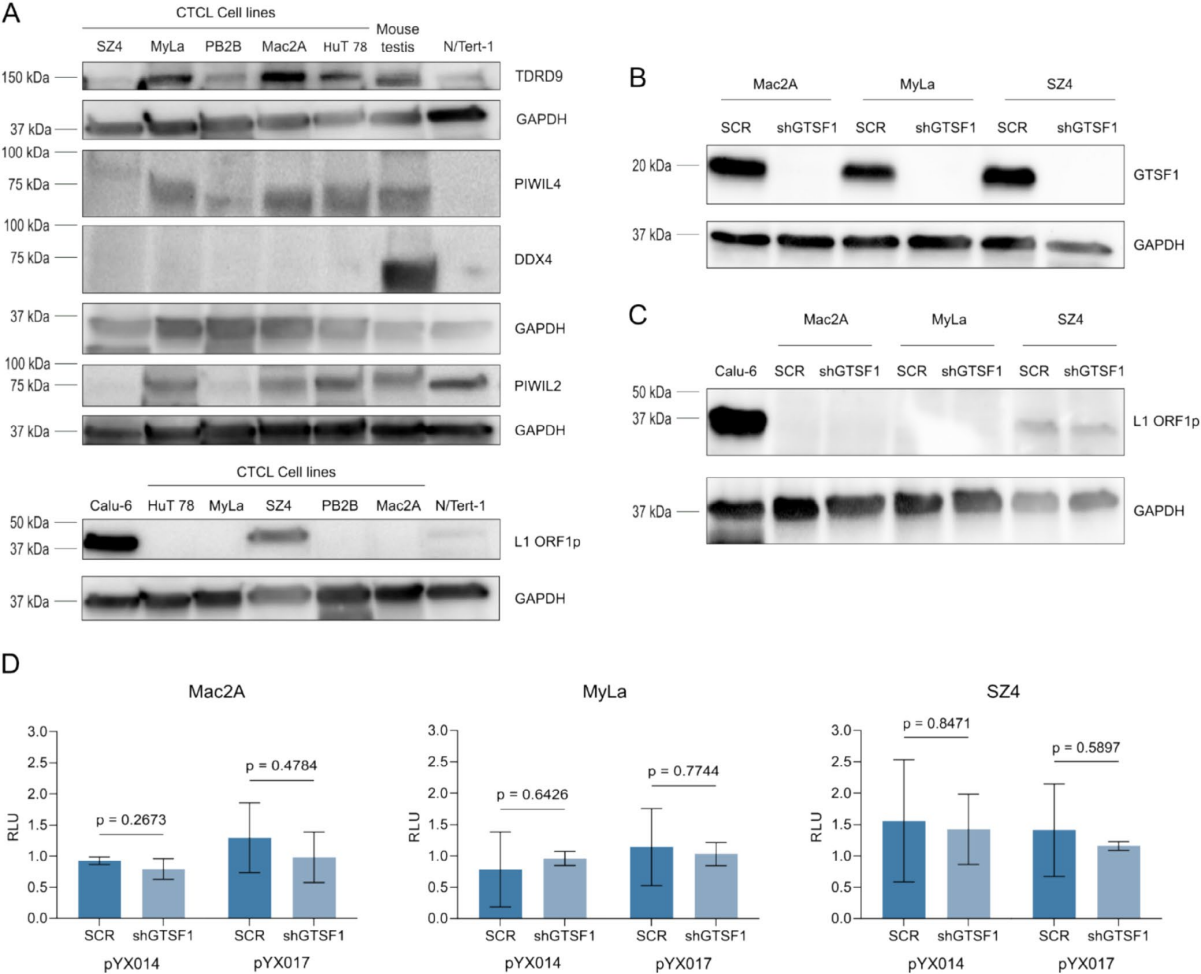
In CTCL, GTSF1 does not regulate retrotransposons. (A) Western blot analysis of piRNA pathway elements TDRD9, PIWIL4, DDX4 and PIWIL2 (top) and of retrotransposon L1 ORF1p (bottom) in CTCL cell lines. Protein from mouse testis is used as a positive control (top), protein from cell line Calu‐6 is used as a positive control (bottom) and protein from cell line N/Tert‐1 (top and bottom) is used as a non‐malignant control. GAPDH was used as a loading control and is presented for each membrane probed. (B) Western blot analysis of GTSF1 after lentiviral shRNA‐mediated knockdown in the three CTCL cell lines Mac2A, MyLa and SZ4. GAPDH was used as a loading control. (C) Western blot analysis of L1 ORF1p after GTSF1 knockdown in CTCL cell lines. Protein from cell line Calu‐6 is used as a positive control. GAPDH was used as a loading control. (D) Luciferase activity from retrotransposition assay in Relative Luminescence Units (RLU). Each assay was performed with two reporter plasmids, pYX014 and pYX017, for each cell line. Firefly luminescence was corrected with the corresponding *Renilla* luminescence value and the retrotransposition incompetent plasmid pYX015 is used to normalise luminescence values. Differences between SCR (dark blue bars) and shGTSF1 (light blue bars) were evaluated with unpaired two‐tailed *t* test. Data are presented as means of three replicates ± SD.

Previous publications reported that GTSF1 participates in the piRNA pathway, silencing retrotransposons during gametogenesis. Therefore, we hypothesised that GTSF1 expression in CTCL was associated with reactivation of this pathway and of retrotransposons. To test this, we analysed expression of other piRNA pathway elements (Figure 2A, top). Western blot of TDRD9, PIWIL4, DDX4 and PIWIL2 demonstrated a heterogeneous expression pattern. Of note, we did not observe expression of DDX4. The absence of DDX4 eliminates the possibility of piRNA pathway reactivation [46]. In addition, to evaluate the reactivation of retrotransposons we tested protein expression of the retrotransposon L1 (Figure 2A, bottom). The L1 protein ORF1p also demonstrated heterogeneous expression. Taken together, these results suggest that the piRNA pathway is not reactivated in selected CTCL cell lines at baseline.

Subsequently, we performed a lentiviral shRNA‐mediated knockdown of GTSF1 in three cell lines. Considering that MF and SS represent > 50% of CTCL cases [47], selecting one cell line representative of each variant along with a cell line of pc‐ALCL would represent the important clinical profiles in CTCL (Figures 2B and S2A,B). After GTSF1 knockdown, we evaluated whether it led to the reactivation or overexpression of retrotransposons. Unexpectedly, GTSF1 knockdown did not lead to increased expression of L1 mRNA (Figure S2C) nor ORF1p (Figure 2C). To evaluate changes in transposition events, we performed a dual‐luciferase retrotransposition assay with two different reporter plasmids, pYX014 and pYX017 [27]. GTSF1 knockdown did not lead to increased luminescence, highlighting that GTSF1 knockdown does not reactivate retrotransposon expression (Figure 1D). Taken together, these data indicate that GTSF1 expression in CTCL is not related to retrotransposon control.

Given the evidence of the role developmental program genes play in carcinogenesis, we evaluated the involvement of GTSF1 in CTCL cells' survival and proliferation. We hypothesised that the ectopic expression of GTSF1 suggests a dependency on this gene. To test this, we performed a cell proliferation assay from 24 h up to 144 h (Figure S3A). GTSF1 knockdown did not affect proliferation nor survival. Due to the limitations associated with this assay, we evaluated apoptosis and proliferation using assays with higher sensitivity.

To evaluate whether GTSF1 knockdown led to increased apoptosis, we performed annexin V/PI staining. We observed no increase in any apoptotic stage (Figure S3B). Then, to evaluate proliferation, we performed immunofluorescence staining for the proliferation marker Ki67. Quantification of Ki67^+^ cells confirmed GTSF1 knockdown did not impact proliferation (Figure S3C). Together, these data suggest GTSF1 is not essential nor provides a selective growth advantage for the survival and proliferation of CTCL cells.

**FIGURE 3:**
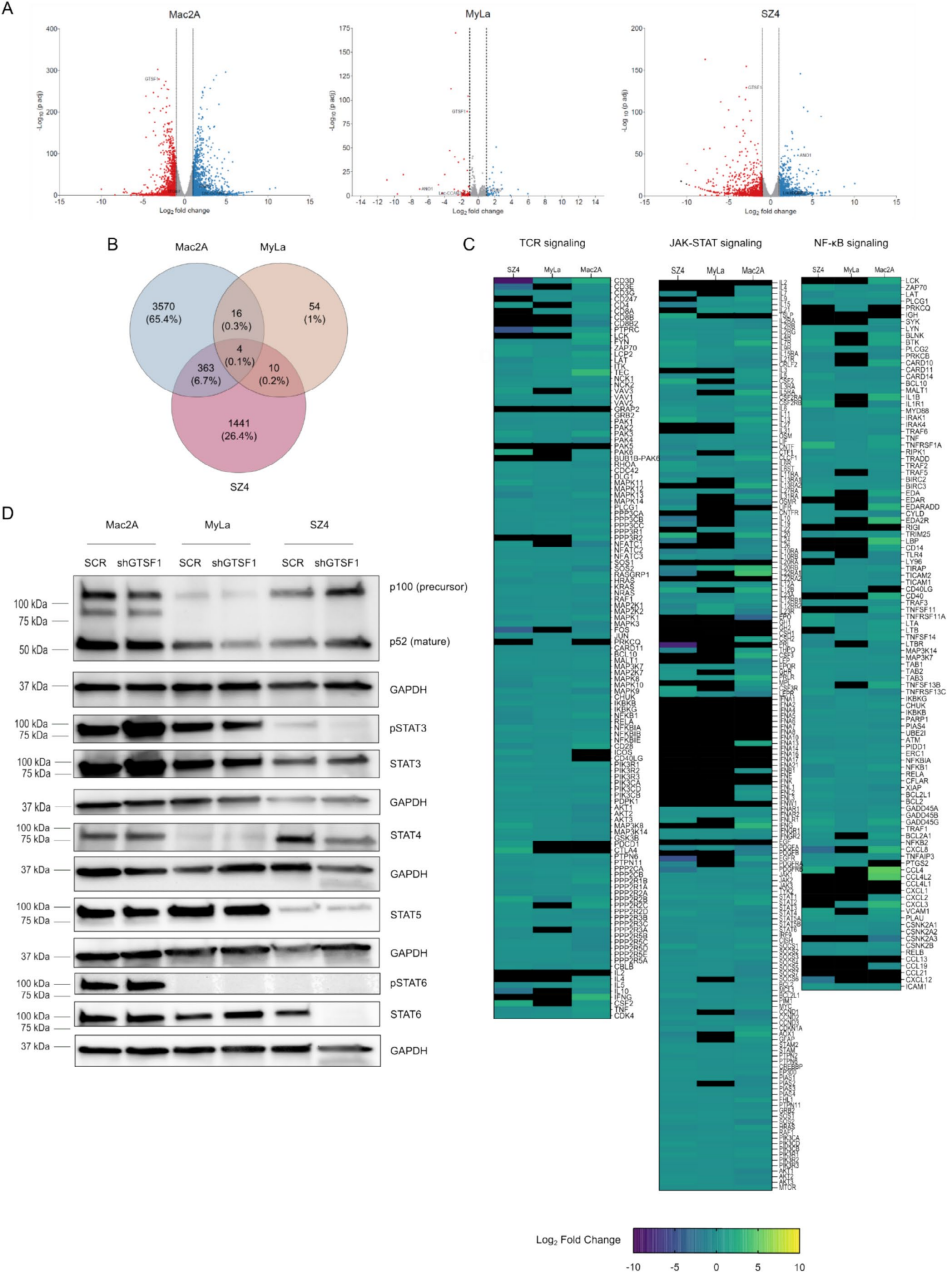
GTSF1 has a different role in each CTCL variant. (A) Volcano plots showing Log_2_ fold change and −Log_10_ (*p*‐adj) after GTSF1 knockdown in CTCL cell lines. Each dot represents one gene. Upregulated genes (Log_2_ fold change ≥ 1) are presented in blue, downregulated genes (Log_2_ fold change ≤ −1) in red and genes with no significant change in grey. GTSF1, ANO1, ITGB7 and Lnc‐CCAR2‐2 are highlighted. (B) Venn diagram of common DEGs (Log_2_ fold change ≥ 1 or (Log_2_ fold change ≤ −1 and p‐adj < 0.05) after GTSF1 knockdown in CTCL cell lines. Percentages in parenthesis are calculated from the total queried genes. (C) Heatmaps showing Log_2_ fold change of gene expression between SCR and shGTSF1 for CTCL cell lines in the following pathways: TCR signalling (left), JAK–STAT signalling (middle), and NF‐κB signalling (right). Each column represents a cell line and each row a gene. Gene lists were retrieved from the KEGG database. The Log_2_ fold change expression scale is presented at the bottom. A black rectangle in the heatmap cell means no expression was detected. (D) Western blot analysis of NFKB2, pSTAT3, STAT3, STAT4, STAT5, pSTAT6 and STAT6 after GTSF1 knockdown in CTCL cell lines. GAPDH was used as a loading control and is presented for each membrane probed.

Considering these findings, we hypothesised the role of GTSF1 to be related to other hallmarks of cancer. To test this, we performed bulk RNA‐Seq and identified DEGs between SCR and shGTSF1 cells (Figure S4). Surprisingly, volcano plots demonstrated markedly different profiles for each cell line (Figure 3A). To assess a common role of GTSF1 across the three cell lines, we created a Venn diagram from the DEGs of the three cell lines and considered the intersection of all three cell lines to increase robustness (Figure 3B). This rendered four genes: GTSF1, validating the knockdown; ANO1, a calcium‐activated channel; ITGB7, a member of the integrin superfamily; and Lnc‐CCAR2‐2, a lnRNA without any previously described role. Importantly, except for GTSF1, none of these genes presented the same direction of dysregulation. Together, these findings suggest that GTSF1 knockdown leads to a different transcriptomic profile for each CTCL variant: This is aligned with the fact that each cell line represents a clinically different entity [45, 48].

To better understand these different profiles, we evaluated the most common dysregulated pathways in CTCL: NF‐κB, JAK–STAT and TCR signalling pathways [49] (Figure 3C). Consistent with earlier findings, each cell line presented a different expression profile for each pathway. Interestingly, Mac2A presented upregulation, MyLa presented both upregulation and downregulation, and SZ4 presented downregulation of genes in these pathways. To further evaluate these different profiles, we performed western blot of selected proteins of the NF‐κB and JAK–STAT pathways (Figure 3D). This confirmed varying expression patterns for each cell line, with a trend of upregulation in Mac2A and downregulation in SZ4. Interestingly, Mac2A showed three NFKB2 bands: the precursor and active forms and a band of approximately 80 kDa. In CTCL, NFKB2 commonly displays C‐terminal deletions rendering the NF‐κB pathway constitutively active [50]. In addition, pSTAT3 and STAT3 showed increased expression after GTSF1 knockdown, suggesting activation of this signalling pathway. Changes in STAT4, STAT5 and STAT6 confirmed downregulation in SZ4. Taken together, these results suggest that the role of GTSF1 is CTCL variant dependent.

**FIGURE 4:**
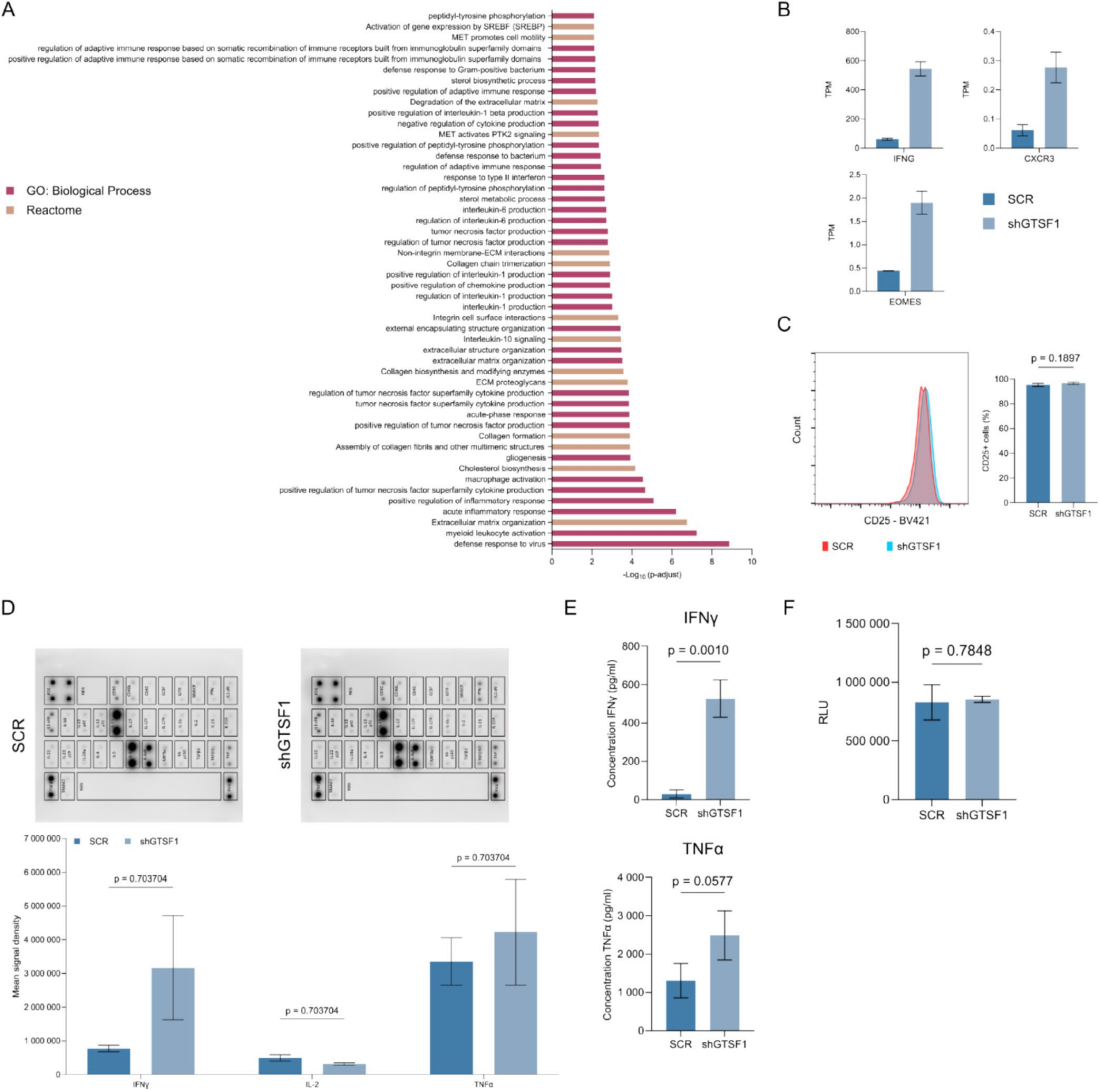
GTSF1 modifies the memory/effector phenotype. (A) Pathway enrichment analysis showing −Log_10_ (*p*‐adj) of the top 50 significantly dysregulated pathways after GTSF1 knockdown in Mac2A. Pathways from Gene Ontology (GO): Biological Processes are shown in pink and pathways from Reactome are shown in brown. (B) Relative expression in Transcripts Per Million (TPM) of selected DEGs in SCR (dark blue) and shGTSF1 (light blue) in Mac2A. Data are presented as means of three replicates ± SD. (C) Flow cytometry analysis of CD25 cell surface marker expression. Representative histogram (left) from SCR (red) and shGTSF1 (blue). Percentage of CD25^+^ (right) cells. Differences between SCR (dark blue) and shGTSF1 (light blue) were evaluated with unpaired two‐tailed *t* test. Data are presented as means of three replicates ± SD. (D) Cytokine production analysis after GTSF1 knockdown in Mac2A. Representative membranes (top) from SCR and shGTSF1 are presented. Mean signal density (bottom) of selected cytokines. Differences between SCR (dark blue bars) and shGTSF1 (light blue bars) were evaluated with Mann–Whitney test and Holm‐Šídák correction method. Data are presented as means of two replicates ± SD. (E) ELISA assays for quantification of IFNγ (top) and TNFα (bottom) production after GTSF1 knockdown in Mac2A. Differences between SCR (dark blue bars) and shGTSF1 (light blue bars) were evaluated with unpaired two‐tailed *t* test. Data are presented as means of three replicates ± SD. (F) Luciferase activity from lactate secretion assay in Relative Luminescence Units (RLU). Differences between SCR (dark blue bars) and shGTSF1 (light blue bars) were evaluated with unpaired two‐tailed *t* test. Data are presented as means of three replicates ± SD.

In CTCL, disease progression is associated with a Th2 phenotype/decreased immune response [51], which contrasts with the immune activation we observed for Mac2A. Given this clinical relevance, we sought to better understand these changes. Pathway enrichment analysis for Mac2A GTSF1 knockdown led to immune activation (Figures 4A and S5A). Top hit pathways included: defence response to virus, myeloid leukocyte activation and acute inflammatory response. Memory and effector T cells behave in a spectrum, with each phenotype at one extreme; movement towards an effector phenotype is caused by activation [52, 53]. Therefore, we evaluated the expression of genes associated with each extreme of the spectrum (Figure 4B). Interestingly, we identified increased expression of genes on both extremes. Knockdown led to increased expression of *IFNG* and *CXCR3*, both associated with effector phenotype and increased expression of *EOMES*, associated with T cell memory phenotype. Together, these findings suggest that GTSF1 knockdown led to a dysregulation in the memory/effector phenotype spectrum.

We then sought to better understand this dysregulation in the memory/effector phenotype. We evaluated activation by quantifying the percentage of CD25^+^ cells as an indicator of activation (Figure 4C). The percentage of CD25^+^ T cells remained unchanged after GTSF1 knockdown. Given that T‐cell activation leads to cytokine production, we evaluated changes in cytokine production with a cytokine membrane. We identified multiple changes in cytokine production; however, none showed statistical significance. Interestingly, GTSF1 knockdown led to an increased production of the Th1‐associated cytokines IFNγ and TNFα. In contrast, IL‐2 showed no change (Figures 4D and S5B). Due to the importance of IFNγ and TNFα in the decline of the immune response in CTCL patients [51], we decided to validate these findings. ELISA assays demonstrated that GTSF1 knockdown led to significantly increased production of IFNγ and increased production of TNFα (Figure 4E). Next, we evaluated metabolic changes associated with the memory/effector T‐cell phenotype. Effector cells rely on glycolysis and production of lactate, while memory cells rely on oxidative phosphorylation [54, 55]. Therefore, we measured extracellular production of lactate (Figure 4F). GTSF1 knockdown did not lead to changes in lactate secretion. This suggests that cells with low GTSF1 expression do not rely on glycolysis and/or production of lactate. Taken together, these data suggest GTSF1 plays a partial role in modifying the memory/effector phenotype in CTCL cells, particularly in the production of cytokines associated with immune activation.

## 4 Discussion

Ectopic expression of germline genes is a well‐established finding in cancer [56]. Despite this, the specific functions many of these genes perform in carcinogenesis remain poorly understood [57]. In CTCL, ectopic expression of GTSF1 and its association with worse clinical prognosis has been consistently reported [8, 9, 10, 11, 12, 13, 24]. Interestingly, ectopic expression of GTSF1 has also been reported in Acute Myeloid Leukaemia [58, 59] and liver cancer [60]. However, the role of GTSF1 in carcinogenesis remains unknown. Here, we aimed to understand its contribution to CTCL carcinogenesis and its impact on malignant T‐cell phenotype.

Previous publications associating GTSF1 expression with a worse prognosis did so by including it as a member of a gene cluster [8, 9, 24]. We demonstrate that for patients in disease stage IIB, GTSF1 expression on its own is associated with accelerated cancer progression and decreased survival—worse overall prognosis. In agreement with previous publications [8, 9, 12, 13, 24], our analysis shows that patients with the most advanced stage of the disease display high GTSF1 expression. Our immunohistochemistry analysis demonstrates a heterogeneous expression pattern in tissues. High genetic/molecular heterogeneity is a well‐established characteristic of CTCL [19, 21, 61]. Therefore, we reason that GTSF1 is an interesting candidate as a progression biomarker for CTCL.

In mouse germline cells, GTSF1 participates in the piRNA pathway, silencing active retrotransposons [16, 62]. Although retrotransposon reactivation and function have been previously reported in CTCL [63], our results indicate that the piRNA pathway is not reactivated. Our results also indicate that GTSF1 knockdown does not lead to an increased rate of retrotransposition events from its baseline. In agreement, previous publications demonstrated that the ectopic expression of other piRNA elements in cancer did not lead to pathway reactivation [46, 64].

Our investigation suggests that in CTCL, GTSF1 partially controls the memory/effector phenotype of the malignant T cells. CTCL arises from skin resident memory T cells [61, 65], which means these cells are inactive. When memory T cells re‐encounter their cognate antigen, they acquire effector phenotype characteristics [54, 66]. GTSF1 knockdown led to T‐cell activation and production of IFNγ and TNFα cytokines. This suggests that GTSF1 knockdown leads to an acquisition of effector phenotype. Although not all effector phenotype markers we evaluated demonstrated a change, this is in line with memory/effector T cells behaving on a spectrum [53, 55, 67]. Interestingly, a recent publication proposed a molecular classification for CTCL based on the malignant T cells and their microenvironment expression profile [11]. The central memory T‐cell profile is associated with GTSF1 expression. Therefore, we speculate that GTSF1 partially modifies the memory/effector phenotype, tilting the balance towards the memory T‐cell phenotype.

Disease progression is associated with a shift in the type of cytokines produced in the skin. In early stage disease, skin samples show production of the Th1 immune‐responsive cytokines IFNγ and TNFα, while in late stages the cytokines produced are the Th2/immune‐repressive IL‐4 and IL‐5 [25, 26]. Our data show the negative role GTSF1 has in controlling the production of cytokines associated with early stage disease. Furthermore, this is mirrored in our patient data analysis, in which patients with high GTSF1 expression demonstrate poor survival. We speculate that GTSF1 modulates early stage cytokines, impacting disease progression.

Here, we present an initial mechanistic study to understand the role of GTSF1 in CTCL. With in vitro approaches, we demonstrate the role of GTSF1 in modifying the memory‐effector phenotype of malignant cells. Studies with in vivo models, other variants of CTCL, and additional patient data will help address this limitation. Specifically, we show that only the cell line Mac2A, representative of pc‐ALCL, responded to GTSF1 silencing. Whether this reflects the mutational status of Mac2A, the variant of CTCL it represents, or even the expression of CD30 is of utmost importance to answer. Furthermore, recent publications have reported that ALCL presents a Th17 profile [68]. In addition to the production of IL‐17, the Th17 profile is also associated with high STAT3 activity and low expression of GATA3 [68]. Our data show that GTSF1 knockdown in Mac2A led to higher transcript expression of *IL17D* and *IL17F* (data not shown) and activation of STAT3; however, GATA3 did not show differential expression. Therefore, future research should consider evaluating the Th17 profile in detail.

Together, our data places GTSF1 as a candidate for a putative disease progression biomarker. Given the heterogeneity that characterises CTCL, its robustness as a biomarker needs to be validated by other groups. Additionally, as a germline gene, GTSF1 has potentially immunogenicity and privileged expression, making it a strong candidate for the development of immunotherapies [57]. We speculate that targeting GTSF1‐expressing cells will allow us to tilt the balance towards the effector phenotype. Favouring an effector phenotype will then lead to an immune‐responsive profile in CTCL patients. Thus, our data suggest that the ectopic expression of germline genes in cancer may allow the malignant cells to recapture the function of these genes in a novel way for their benefit. Understanding the role these genes play in carcinogenesis can help us discern the best candidates for novel treatments.

## Author Contributions

A.M.V. designed research, performed experiments, analysed and interpreted data, performed statistical analysis and wrote the manuscript. J.G. designed research and performed experiments. P.X. and P.L. analysed data and performed statistical analysis. B.R. edited the manuscript. I.V.L. designed research, analysed and interpreted data, provided resources and supervised the study. All authors have read and agreed to the published version of the manuscript.

## Ethics Statement

All patients were enrolled in this study with written informed consent and in accordance with the Declaration of Helsinki. Samples were obtained after approval from The Ottawa Hospital Research Institute (REB study #20150896‐01H), Research Institute of the McGill University Health Centre and affiliated hospitals (REB study #A09‐M81‐10A) and Laval University (REB study # 2011HES‐22808). The patient cohort has been previously described [9]. Samples were obtained by punch biopsy of the lesional skin and processed into formalin‐fixed, paraffin‐embedded (FFPE) tissue blocks.

## Conflicts of Interest

The authors declare no conflicts of interest.

## Supporting information


**Figure S1.** Heterogeneous GTSF1 expression in cancer and CTCL. (A) Survival (Kaplan–Meier) plots of CTCL patients’ disease outcomes stratified by stage IIB and IVA2. Differential survival between high (brown bars) and low (pink bars) *GTSF1* expression groups was identified with Log‐rank (Mantel‐Cox) test with *p* < 0.05 (B) Immunohistochemistry of GTSF1 in skin biopsies from CTCL patients. Each panel represents a different patient. Arrows indicate nuclear expression of GTSF1 in pleomorphic epidermotropic lymphocytes. Negative and positive controls are presented in the main figure and an additional negative control, benign dermatitis, is presented at the bottom. Scale bars represent 50 μm. (C) Relative *GTSF1* expression normalised to *ACTB* in a panel of CTCL cell lines. Expression is normalised to the highest‐expressing cell line. Data are presented as means ± SD.
**Figure S2.** In CTCL, GTSF1 does not regulate retrotransposons. (A) Western blot analysis of GTSF1 after lentiviral shRNA‐mediated knockdown with three different clones in Mac2A and MyLa cell lines. GAPDH was used as a loading control. The clone V2LHS_24307 was used for subsequent experiments. (B) Relative *GTSF1* expression normalised to *GAPDH* after GTSF1 knockdown in SCR (dark blue) and shGTSF1 (light blue) in CTCL cell lines. Expression is normalised to SCR of each cell line. Data are presented as means ± SD. (C) Relative ORF1 (left) and ORF2 (right) mRNA expression normalised to *GAPDH* after GTSF1 knockdown in Mac2A. Expression is normalised to SCR. Differences between SCR (dark blue) and shGTSF1 (light blue) were evaluated with unpaired two‐tailed *t* test. Data are presented as means of three replicates ± SD.
**Figure S3.** GTSF1 is not essential for CTCL cells survival. (A) Cell proliferation assay from 24 h up to 144 h comparing SCR (dark blue bars) and shGTSF1 (light blue bars). Data are presented as mean cell numbers of three replicates ± SD. (B) Apoptosis assay using flow cytometry. Representative dot plots (left top) from SCR and shGTSF1 MyLa are presented. Percentage of total apoptotic cells (right top) defined as annexin V^+^ + annexin V^+^ PI^+^ cells. Percentage of early apoptotic cells (left bottom) defined as annexin V^+^ cells. Percentage of late apoptotic cells (right bottom) defined as annexin V^+^ PI^+^ cells. Differences between SCR (dark blue) and shGTSF1 (light blue) were evaluated with unpaired two‐tailed *t* test. Data are presented as means of three replicates ± SD. (C) Immunofluorescence staining and quantification for proliferation marker Ki67. Percentage of proliferating cells (left) defined as Ki67 positive. Differences between SCR (dark blue bars) and shGTSF1 (light blue bars) were evaluated with unpaired two‐tailed *t* test. Data are presented as means of three replicates ± SD. Representative photos of DAPI, Ki67 and merged channels (right) from cell line Myla are presented. Scale bars represent 20 μm.
**Figure S4.** GTSF1 has a different role in each CTCL variant. (A) Principal Component Analysis (PCA) of the normalised RNA‐Seq data after GTSF1 knockdown in CTCL cell lines.
**Figure S5.** GTSF1 modifies the memory/effector phenotype. (A) Pathway enrichment analysis showing −Log_10_ (*p*‐adj) of the top 50 significantly dysregulated pathways with DEGs after GTSF1 knockdown in SZ4. Pathways from Gene Ontology (GO): Biological Processes are shown in pink and pathways from Reactome are shown in brown. (B) Cytokine production analysis after GTSF1 knockdown in Mac2A. Mean signal density (bottom) of additional cytokines. Differences between SCR (dark blue) and shGTSF1 (light blue) were evaluated with Mann–Whitney test and Holm–Šídák correction method. All comparisons resulted *p*‐adj ≥ 0.99 . Data are presented as means of two replicates ± SD.

## Data Availability

RNA‐Seq data have been deposited in Gene Expression Omnibus database under accession number GSE270818. Original data sets generated and/or analysed here and not available in GEO are available on request from first author Amelia Martinez Villarreal (amelia.martinezvillarreal@mail.mcgill.ca).
