# Supplementary figures and images for: Memory T‐Cell Phenotype in Cutaneous T‐Cell Lymphoma Is Modified by Germline Gene Gametocyte Specific Factor 1
Source: Exp Dermatol. 2025 May 14;34(5):e70123. doi: 10.1111/exd.70123 (PMC12078864; doi:10.1111/exd.70123)

**SUPPLEMENTARY FIGURES**


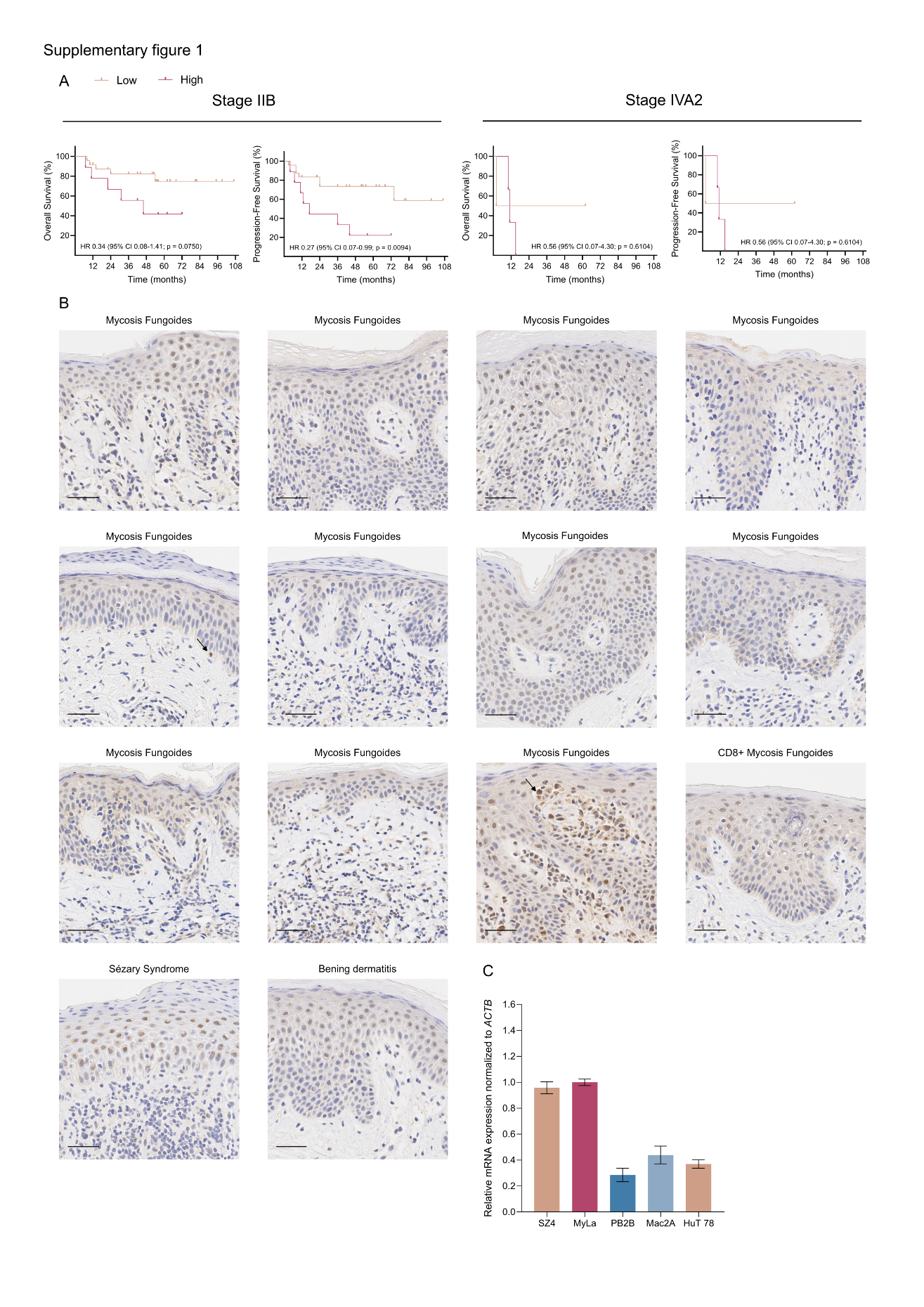


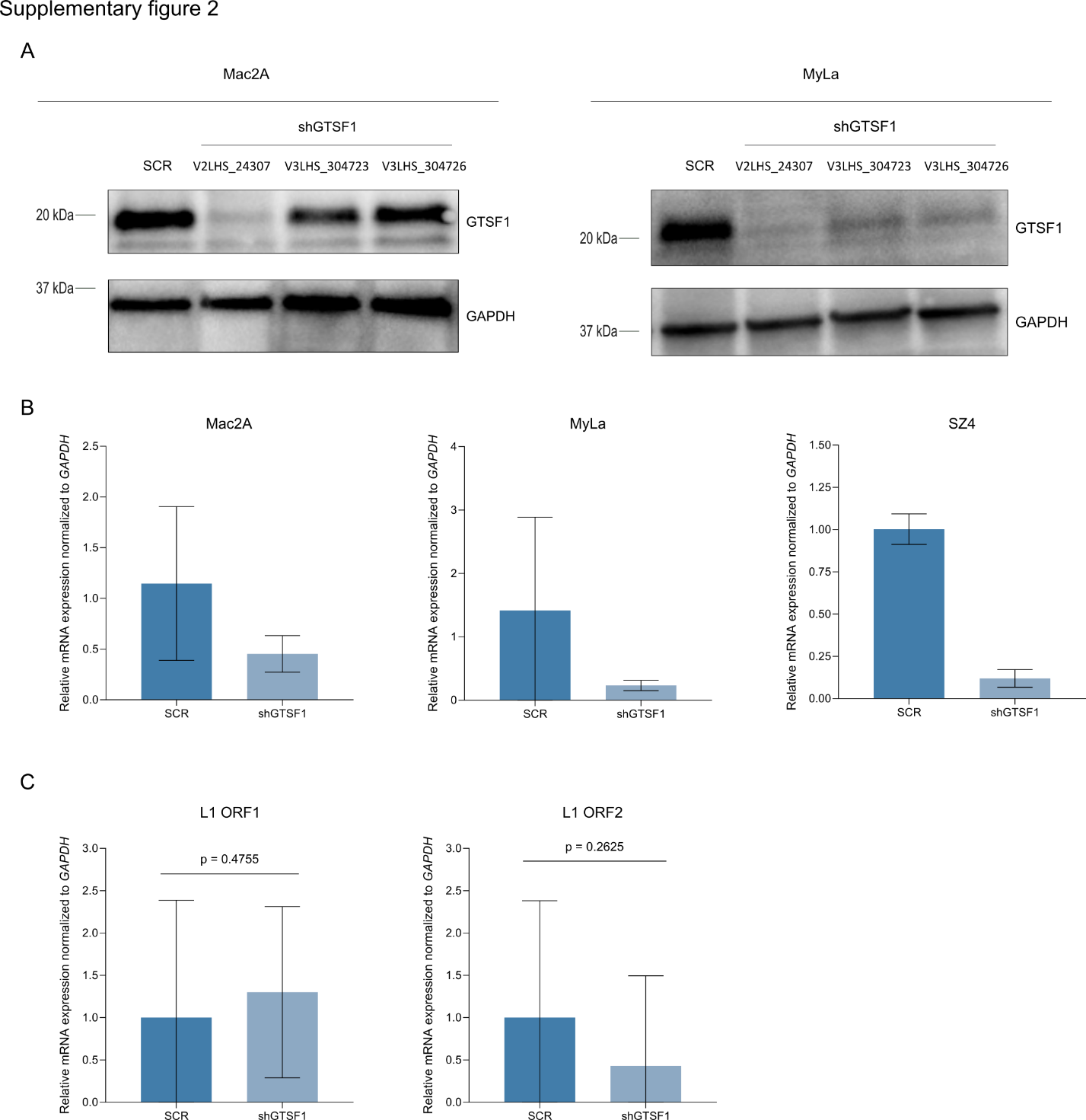


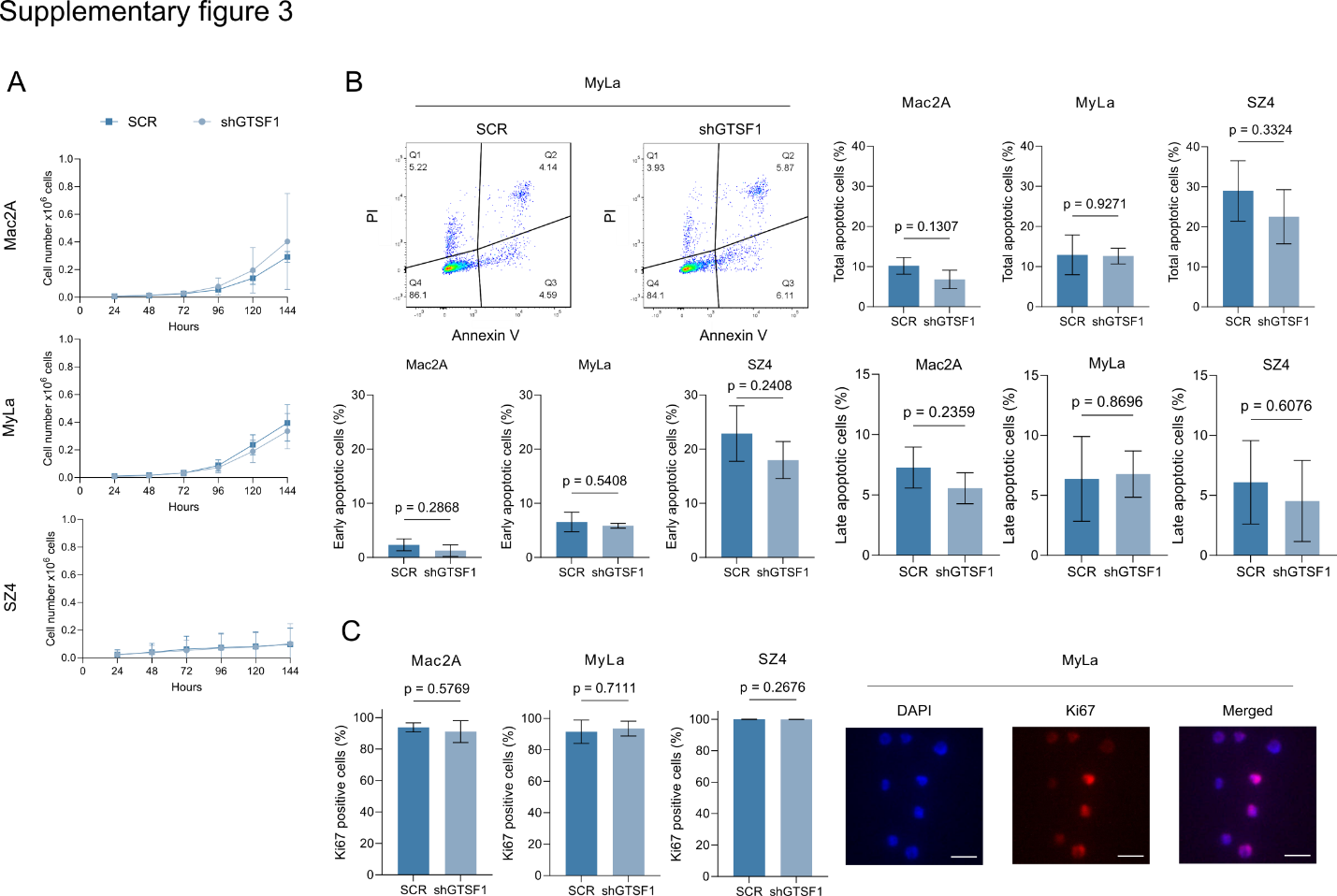


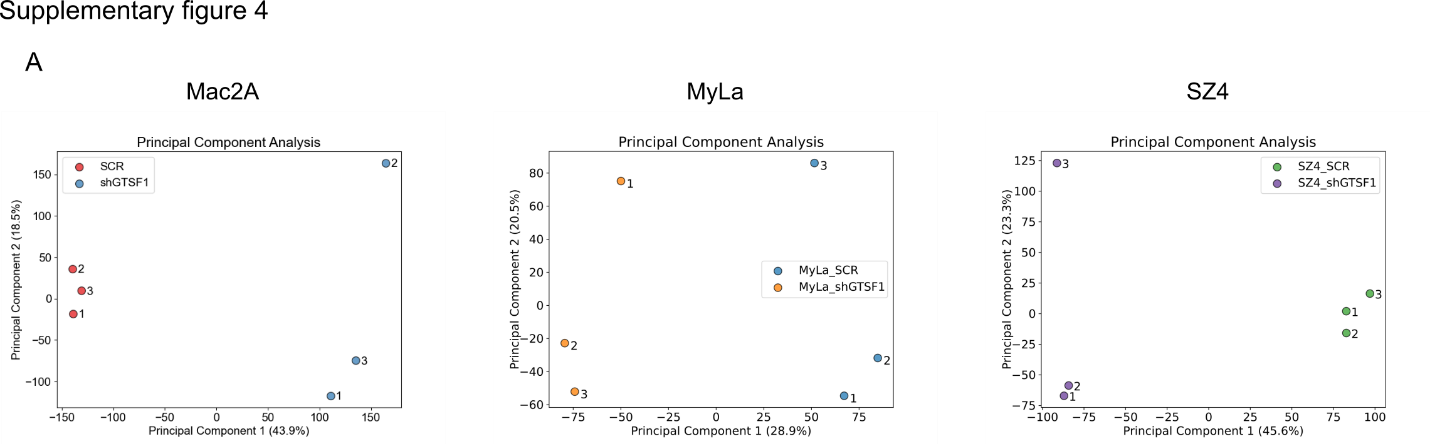


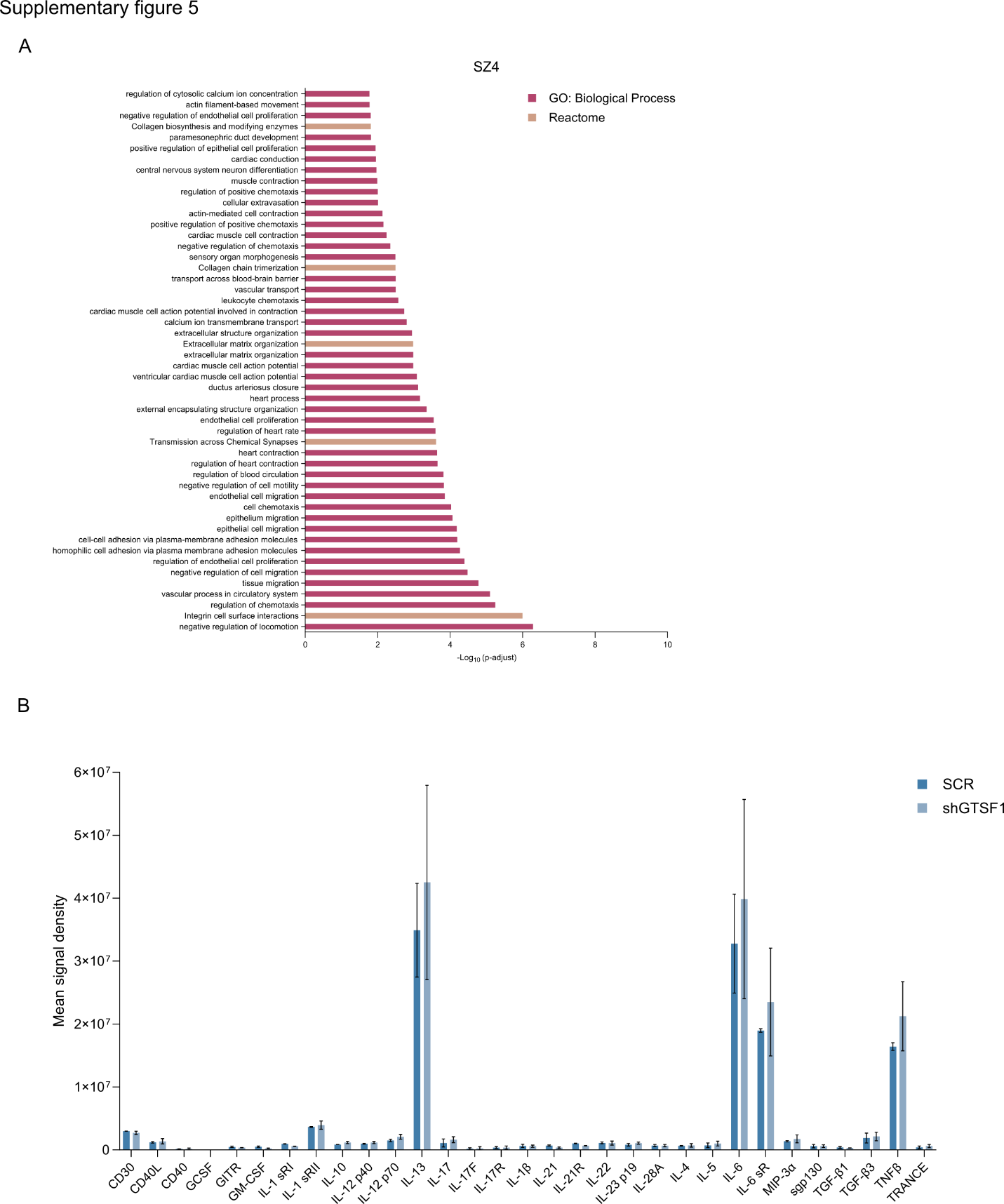

Supplement: Supplementary file 1 — Figure S1. Heterogeneous GTSF1 expression in cancer and CTCL. (A) Survival (Kaplan–Meier) plots of CTCL patients’ disease outcomes stratified by stage IIB and IVA2. Differential survival between high (brown bars) and low (pink bars) GTSF1 expression groups was identified with Log‐rank (Mantel‐Cox) test with p < 0.05 (B) Immunohistochemistry of GTSF1 in skin biopsies from CTCL patients. Each panel represents a different patient. Arrows indicate nuclear expression of GTSF1 in pleomorphic epidermotropic lymphocytes. Negative and positive controls are presented in the main figure and an additional negative control, benign dermatitis, is presented at the bottom. Scale bars represent 50 μm. (C) Relative GTSF1 expression normalised to ACTB in a panel of CTCL cell lines. Expression is normalised to the highest‐expressing cell line. Data are presented as means ± SD. Figure S2. In CTCL, GTSF1 does not regulate retrotransposons. (A) Western blot analysis of GTSF1 after lentiviral shRNA‐mediated knockdown with three different clones in Mac2A and MyLa cell lines. GAPDH was used as a loading control. The clone V2LHS_24307 was used for subsequent experiments. (B) Relative GTSF1 expression normalised to GAPDH after GTSF1 knockdown in SCR (dark blue) and shGTSF1 (light blue) in CTCL cell lines. Expression is normalised to SCR of each cell line. Data are presented as means ± SD. (C) Relative ORF1 (left) and ORF2 (right) mRNA expression normalised to GAPDH after GTSF1 knockdown in Mac2A. Expression is normalised to SCR. Differences between SCR (dark blue) and shGTSF1 (light blue) were evaluated with unpaired two‐tailed t test. Data are presented as means of three replicates ± SD. Figure S3. GTSF1 is not essential for CTCL cells survival. (A) Cell proliferation assay from 24 h up to 144 h comparing SCR (dark blue bars) and shGTSF1 (light blue bars). Data are presented as mean cell numbers of three replicates ± SD. (B) Apoptosis assay using flow cytometry. Representative dot plots (left [file EXD-34-e70123-s001.docx]
